# Creating Our Professional Evidence‐Base: A Journey, Not a Destination

**DOI:** 10.1002/jmrs.70003

**Published:** 2025-06-23

**Authors:** Jonathan P. McNulty

**Affiliations:** ^1^ Radiography and Diagnostic Imaging School of Medicine, University College Dublin Dublin Ireland

## Abstract

Let's continue this essential and exciting journey as a profession and then we can celebrate our achievements, celebrate our new collaborations, and celebrate the impact of our research!

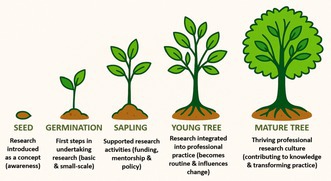

Sackett et al. [1] defined evidence‐based practice (EBP) as:

“The conscientious, explicit and judicious use of current best evidence in making decisions about the care of the individual patient ……… integrating individual clinical expertise with the best available external clinical evidence from systematic research.”

The two core components in this are the ‘best research evidence’ and ‘individual clinical expertise’. Historically, the ‘clinical expertise’ building this evidence‐base was often driven by radiologists, radiation oncologists, nuclear medicine physicians, and medical physicists. It is still the case today that many publications arising from research directly linked to the day‐to‐day professional roles and responsibilities of radiographers/medical radiation technologists, have authorship teams with no radiographers involved. We thus need to add our own ‘professional expertise’ as radiographers/medical radiation technologists. Our contribution as radiographers to this evidence, through undertaking quality research and subsequent dissemination, is essential and will also serve to raise the profile and standing of radiography beyond our profession. We need to move from being the facilitators of research for other professions, to being equal partners and leaders in research. We must be the ones driving forward the research directly linked to our roles and responsibilities as we work to build and strengthen our own professional evidence‐base.

The World Health Organization (WHO) defines health professionals as those who “maintain health in humans through the application of the principles and procedures of evidence‐based medicine and caring” and the definition goes on to state that health professionals “also conduct research and improve or develop concepts, theories and operational methods to advance evidence‐based health care” [2]. So, if we do not believe in EBP, we should not call ourselves health professionals. Should further motivation be required on why we must grow our professional evidence‐base, and in turn raise the wider profile of our profession, remember that the International Labour Organization (ILO) does not currently recognise radiographers/medical radiation technologists as ‘professionals’ (ILO International Standard Classification of Occupations (ISCO) major group 2). Rather, we are recognised internationally in ISCO major group 3 as ‘technicians and associate professionals’. In Europe, after many years of lobbying by the European Federation of Radiographer Societies (EFRS), in 2018 our profession, was elevated from ISCO major group 3 to major group 2 ‘professionals’ by the European Commission's European Skills/Competences, qualifications and Occupations (ESCO) unit. Unfortunately, not all is perfect in Europe as in 2022, the occupation ‘radiation therapist’ was added to the ESCO listing, however, in the efforts to add this occupation, radiation therapists are now left in major group 3 ‘technicians and associate professionals’.

All research must start somewhere, as we must as researchers or clinicians engaging with research, and likewise the creation of a professional research culture. We are fortunate to have some hubs of significant research activity involving our profession, with some examples of countries standing out in relation to this; but for the most part, such activity maps back to single institutions and a handful of very active researchers. Common barriers to building a professional research culture are often described internationally, but common opportunities may allow us to replicate initiatives to progress research engagement through learning from each other and through international collaboration. Over the past 20 years, there has been clear growth in the quantity and quality of radiography research internationally; however, we are still a long way from our destination. In some centres and countries, radiographers/medical radiation technologists are taking their first steps engaging in research; however, more support and mentorship is needed across all countries to allow us to undertake more impactful research which enhances our profession and helps transform patient care. Our journey as a profession continues, and we must never become complacent. We need more research activity at all levels, and we need to be even more conscious of the quality of our research. While the seeds of building our professional research culture have long been planted, are we at the sapling stage or the young tree stage, as illustrated in Figure 1 ? As a profession, we certainly have more years to grow before we are mature.

**FIGURE 1:**
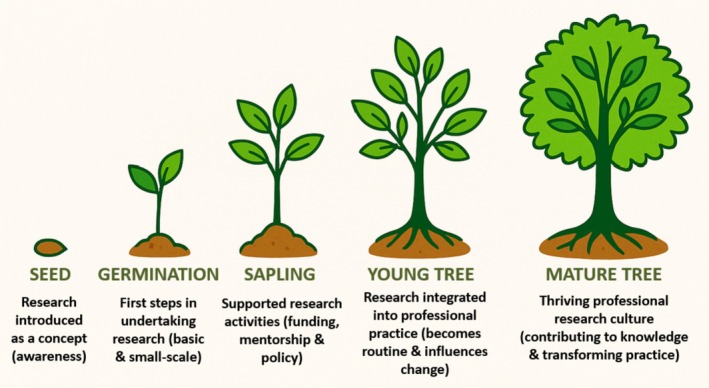
The development of a professional research culture.

The recent article by Li and Mdletshe exploring New Zealand's position in the world of radiology, nuclear medicine, and medical imaging research output discusses several points which, as a profession, we must all reflect on [3]. They compare New Zealand to Australia, the United States of America (USA), the United Kingdom (UK), Canada, Ireland, and South Africa [1]. It would be interesting to look beyond this selection of countries to include others who have strategically worked to grow medical radiation sciences research to see where they sit.

Li and Mdletshe report the annual publication growth for each of the included countries [3], and the growth aligns with the 4% annual growth across scientific publications in general which was reported in 2021 [4]. The absence of a significant spike in publications for 2020 and 2021, which occurred during the first year of the pandemic, was not evident. Indeed, Nane et al. reported a growth of 846% from May 2020 to May 2021, across scientific publications [5]. It is thus somewhat unexpected not to see this growth in our field.

When it comes to citations between 1996 and 2022, the data are interesting in the context of self‐citations, with authors from the USA more likely to cite their own work (44.8% self‐citations) versus those in New Zealand (10.0% self‐citations), with Irish authors having the lowest self‐citations at 7.6% [3]. What is it about the publications from New Zealand that makes them the leaders in terms of the percentage of documents cited at 77.5%? What are the factors influencing this, and can these significant differences be explained?

International and industry collaborations are always referenced in terms of growing our research and making it more impactful. Li and Mdletshe [3] also discuss this, and it would be interesting to explore this further to consider factors which may influence the level of such collaborations—for example, could New Zealand's location on the globe be a barrier to international and industry collaboration?

The USA dominates across metrics evaluated by Li and Mdletshe and suggests the scale of the US scientific community, in this case the radiology, nuclear medicine, and medical imaging community, and the availability of significant research funding [3] as potential influencers. Perhaps the impact of recent changes in the USA, with active research grants being cancelled, new funding opportunities being slashed, and talk of leading academics being forced to leave the US to continue their research [6], will change this data, and the position of the USA, when viewed again in future?

A weak research culture, a low number of doctoral qualifications in the profession, and the absence of research strategies or a national professional research committee are cited as factors influencing New Zealand's ranking across many of the metrics. If you look at radiographers/medical radiation technologists in Ireland, Canada, the USA, and South Africa, many of these points also hold true. In general, these are areas almost every country must work on through our national professional bodies and universities. Clearly recognising research and EBP within the professional requirements, standards of proficiency, and strategy of regulators and professional bodies is a must!

The relative ratio of the radiographers/medical radiation technologists to radiologists, radiation oncologists, nuclear medicine physicians, and medical physicists, in each country is another factor to consider. These ratios can influence many aspects of what the respective professionals do in terms of their daily roles and responsibilities, including their likelihood to engage in research activity. The level of education available in a country—for example, diploma, bachelors, masters, and not just doctoral qualifications—is also an influencing factor.

As well as recognising the need for further follow‐on research, Li and Mdletshe highlight the need to build research networks and collaborations, nationally but especially internationally. They also emphasise the need to further develop research skills within curricula to develop the evidence‐based practitioners of the future, to establish a dedicated national research committee, and building a research culture [3]. While the current paper compares New Zealand to other countries, including four that the authors identify as ‘research leaders’, all countries and jurisdictions have work to do as we work toward maturity (Figure 1). As part of a Delphi‐based project to set out a vision for the future of our profession, the EFRS Radiographer Education, Research, and Practice Project [7], included 11 statements related to our professional expectations and ambitions to aim for by 2031 were established. These statements of expectations and ambitions provide a framework to work toward to establish a mature professional research culture, such as:

- Research being part of the scope of practice of all radiographers/medical radiation technologists as critical users of research findings, as contributors to research teams, or as leaders of research projects.
- Radiographers/medical radiation technologists taking charge of evaluating and developing their practice and the contributions they make to the health and well‐being of patients, communities, and the public.
- The prioritisation of research where potential outcomes are likely to provide positive value for the quality of care delivered to patients and the public.

While focused on Europe, I would encourage all to read and reflect on these research expectations and ambitions and to use them to help inform strategic plans linked to growing and nurturing our professional research culture.

As a profession we need to share and develop best practice, promote research across our profession, inspire the next generation of evidence‐based practitioners, and provide support, mentorship, and encouragement for research. By developing our research culture, expanding collaborations, translating our research into practice, and enhancing our dissemination, we will start to see the impact of EBP in terms of professional advancement, improved patient safety, efficiencies in service delivery, improved patient outcomes, and thus improved quality of life. To do this, we also need to be conscious of the type of research we are undertaking, moving up the hierarchy of research evidence from lower level/less rigorous research to higher level/rigorous/more impactful research [8], especially when 42% of what is published across this journal, the *Journal of Medical Radiation Sciences*, the *Journal of Medical Imaging and Radiation Sciences*, and *Radiography* are descriptive cross‐sectional survey studies and narrative reviews [9].

So, let us continue this essential and exciting journey as a profession and then we can celebrate our achievements, celebrate our new collaborations, and celebrate the impact of our research!

## Conflicts of Interest

J.P.M. is the current Editor in Chief of *Radiography* and is a past president of the European Federation of Radiographer Societies.

## Data Availability

Data sharing is not applicable to this article, as no new datasets were generated or analysed.

## References

[1] D. L. Sackett , W. M. C. Rosenberg , J. A. M. Gray , R. B. Haynes , and W. S. Richardson , “Evidence Based Medicine: What It Is and What It Isn't,” BMJ 312 (1996): 71–72, 10.1136/bmj.312.7023.71. 8555924 PMC2349778

[2] World Health Organization , Transforming and Scaling up Health Professionals' Education and Training: World Health Organization Guidelines 2013 (World Health Organization, 2013). 26042324

[3] V. Li and S. Mdletshe , “Evaluation of New Zealand's Radiology, Nuclear Medicine, and Medical Imaging Research Output: A Bibliometric‐Based Approach,” Journal of Medical Radiation Sciences 72 (2025): 315–324, 10.1002/jmrs.875. PMC12420660 40007131

[4] L. Bornmann , R. Haunschild , and R. Mutz , “Growth Rates of Modern Science: A Latent Piecewise Growth Curve Approach to Model Publication Numbers From Established and New Literature Databases,” Humanities and Social Sciences Communications 8, no. 1 (2021): 1–15, 10.1057/s41599-021-00903-w.38617731

[5] G. F. Nane , N. Robinson‐Garcia , F. van Schalkwyk , and D. Torres‐Salinas , “COVID‐19 and the Scientific Publishing System: Growth, Open Access and Scientific Fields,” Scientometrics 128, no. 1 (2023): 345–362, 10.1007/s11192-022-04536-x. 36246788 PMC9548429

[6] J. Tollefson , D. Garisto , M. Kozlov , and A. Witze , Trump Proposes Unprecedented Budget Cuts to US Science, Nature News, https://www.nature.com/articles/d41586‐025‐01397‐1. 10.1038/d41586-025-01397-1 40316851

[7] European Federation of Radiographer Societies , EFRS White Paper on the Future of the Profession. Radiographer Education, Research, and Practice (RERP): 2021–2031 (Utrecht: European Federation of Radiographer Societies, 2021), https://efrs.eu/publications.

[8] C. Kilgour and L. J. Delaney , “Hierarchy of Research Evidence,” in Quality in Healthcare: Assessing What We Do (University of Queensland, 2024), 10.14264/4174bad.

[9] E. Iweka , B. N. Ezenwuba , and B. Snaith , “Research Designs of Publications in Radiography Professional Journals—A Modified Bibliometric Analysis,” Radiography 30, no. 4 (2024): 1210–1218, 10.1016/j.radi.2024.06.005. 38905765

